# Experiences of a lived experience recovery organisation for those in abstinence-based substance use recovery: a thematic analysis

**DOI:** 10.1186/s13011-025-00671-9

**Published:** 2025-10-09

**Authors:** Gabrielle Humphreys, Natalie Finch

**Affiliations:** 1 Department of Psychology, School of Health and Society, University of Salford, Allerton Building, Salford, M6 6PU United Kingdom; 2 Department of Nursing, School of Nursing and Healthcare Leadership, University of Bradford, Richmond Building, Bradford, BD7 1DS United Kingdom

**Keywords:** Substance use, Addiction, Lived experience, Peer support, LERO, Treatment, Recovery

## Abstract

**Background:**

Lived experience recovery organisations (LEROs) are social support services facilitated by those who have shared lived experience. Typically, they aim to build shared identity and reducing stigma in this area, although there is limited knowledge on the experiences of those using LEROs, with research rarely permitted into these groups. The current study aims to provide insight into these groups, examining the experiences of service users in a UK-based LERO focussed on substance use disorder recovery.

**Methods:**

Fifteen service users were interviewed about their experiences attending this LERO. Transcripts from these semi-structured interviews were thematically analysed by authors, with an inductive approach adopted.

**Results:**

Eight themes and 10 sub-themes were identified. Themes were; Feeling supported in recovery, Experiencing life outside of substance use disorder, Fun, Skills acquisition, Preventing relapse by filling time, Gaining a sense of community, Psychological impact, and Changes in public perception. Participants reported having a positive experience within this LERO, particularly in comparison to traditional treatment pathways. Specifically, participants highlighted feelings of self-worth, belongingness, and enjoyment from this LERO – experiences they felt made this treatment pathway unique.

**Conclusion:**

This paper highlighted the importance of peer support in substance use disorder recovery. Embedding those with lived experience into services was highly valued by participants and generated a unique culture of comfort, hope and opportunity. Although the scope of this study was limited to participants only currently attending this organisation, those interviewed significantly valued this LERO, highlighting their future potential to alleviate the lack of satisfaction reported by some around traditional treatment methods.

**Supplementary Information:**

The online version contains supplementary material available at 10.1186/s13011-025-00671-9.

## Background

### Prevalence of drug and alcohol use

During the last year, 8.8% of individuals in England and Wales reported having used drugs, equating to over 2.9 million people [26]. While many individuals will use drugs and alcohol recreationally, 290,635 adults engaged with NHS drug and alcohol treatment services from April 2022 to March 2023. Within England and Wales 5,448 deaths were recorded as related to drug poisoning—one of the highest drug-induced death rates in Europe and a figure which is steadily increasing – suggesting that current interventions may not be preventing the most serious harm [27, 35]. Furthermore, within individuals using drug and alcohol services in England and Wales, 20% of service users reported having no home and 9% reported a risk of homelessness in the next eight weeks. Additionally, the link between addiction and mental illness is well established, with 71% of those using alcohol and drug services in England and Wales also reporting the need for mental health support [9, 15].

### Traditional approaches to addiction treatment

When presenting to healthcare settings for treatment of substance use disorder, people should be assessed by suitability trained practitioners using appropriate assessment tools. Subsequently, a person-centred package of care developed in collaboration with the patient which meets their individual circumstances and treatment goals should be offered. This may consist of a combination of pharmacological and psychosocial interventions, which must be delivered by competent practitioners who are also able to assess the efficacy of the interventions through treatment, either in the community or an inpatient setting [25]. However, the gap between ideal treatment pathways and real-world practice within healthcare settings must be acknowledged. With the NHS facing a workforce crisis in terms of recruitment struggles, high staff turnover, and long-term absences, the ‘quantity, quality, and morale’ of the sector has declined. As a result, this gold standard of care is unfortunately rarely reached.

In addition, or as an alternative to, this healthcare focused treatment pathway many individuals choose to attend abstinence-based, peer support groups to achieve and subsequently maintain abstinence and prevent relapse. With these challenges in mind, social support groups including LEROs may improve service user experiences by offering consistent, peer-led sessions that compliment overstretched traditional services. The role of these groups has been highlighted as in the UK clinical guidelines for alcohol treatment, with it noted that ‘peers and recovery communities can play a central role in supporting recovery’. These psychosocial interventions were labelled as ‘an essential part of treatment’ and should be collaborative between both experts by profession and experts by lived experience.

### The role of social support in recovery

Social support services are a popular option for individuals in the UK, with Day et al.’s [8] UK National Recovery Survey showing that over half of those in assisted treatment preferred less formal settings, such as mutual help groups (e.g. Alcoholics Anonymous, SMART Recovery, 29.7%) or community-based recovery support groups (e.g. recovery-led community centres; 22.6%). Tracy and Wallace’s [36] systematic review highlighted the benefits of peer support groups with increased treatment engagement, promotion of self-efficacy and coping skills, and a reduction in substance use and risky behaviours concluded. With high rates of service user dissatisfaction, relapse, and attrition in traditional addiction treatment, Kulik and Shah [18] highlighted how peer support worker led activities could increase patient satisfaction within a UK specialist addiction unit. Notably, the simple and low cost activity of running a breakfast club brought the most change in patient satisfaction,a relatively simple event to implement. This intervention may have resulted in positive outcomes due to increases in perceived social connectedness; a factor accounting for 14% of the variance in quality of life scores in Bathish et al.’s [3] study. Stanojlović and Davidson [34] similarly highlighted how peer recovery support services (PRSS) could compliment traditional services by addressing these common issues, although this was in a US sample.

Alexander’s [1] theory of dislocation proposes that substance use disorder is linked to detachment and a loss of identity, holding similar implications for social treatment and prevention of substance use disorder [21]. This theory is supported Pickard’s [28] research, in which the presence of rich social relationships – the opposite of dislocation – were a mediator for treatment success. However, this relationship is not clear cut. For example, found a strong sense of identity and social circle present in individuals during continued heroin use, reporting these participants as ‘far from becoming socially isolated’, and thus, emphasising that dislocation is not always a factor in drug use. Rather than dislocation, participants reported the formation of new in-groups with shared goals around substance use, aligning with Social Identity Theory [4].

While theoretical debates remain, and it is unlikely one framework can singlehandedly explain the complex behaviour of addiction, these findings collectively highlight the importance of social interventions within substance use disorder recovery. These social interventions can assist individuals in navigating identity shifts and social transitions which occur during recovery [4, 31]. Social support groups are also crucial for promoting motivation and capability [31], mechanisms crucial for long-term behaviour change [22]. Furthermore, social support groups can also support individuals after formal treatment has ended, with a social aftercare programme found to further reinforce motivation and structure life post-treatment [30]. The addition of peer support services has been deemed as useful for all stages of recovery in the recent recommendations by reducing harm reduction, increasing service engagement, and sustaining recovery via the development of social capital [33].

### Lived experience recovery organisations

Some recovery support services are facilitated by those with lived experience of substance use disorder, coined Lived Experience Recovery Organisations (LEROs). LEROs are known for the deliverance of activities which aim to promote social identity outside of substance use which are facilitated only by those who have had lived experience in this area. Groups are typically known for their focus on stigma-reduction and advocacy around substance use disorder. With over 50 LEROs currently registered in the UK, these range from small individual projects in a local area to expanded organisations with formal structures. Across all domains of healthcare, there is an increasing expectation that experts by experience are embedded within service provision [32]. The importance of doing so was further emphasised in Dame Carol Black’s Review of Drugs: phase one report, with Black advocating for the integration of peer intervention throughout the entirety of treatment journey. The credibility and authenticity of these LEROs were recognised, as well as their opportunity to challenge stigma, both within society and within healthcare provision [7]. The integration of those with lived experience into healthcare services has been shown to play a crucial role within the facilitation of traditional treatment,acting as a safe, unbiased and trustworthy person for a service users [6]. Furthermore, the discussion between two experts by experience may not be replicable by a healthcare professional due to the incredibly in-depth and unique experience of addiction and change in social identity [4, 31]. In their systematic review examining the role of experts by experience in clinical settings, concluded that this addition of mentors with lived experience led to lower relapses of substance use, increased service user satisfaction and improved relationship formation between service user and clinician. Fallin-Bennett, Elswick and Ashford [12] also studied the impact of lived experience peer support, focussing on the role of peer support specialists (PSSs) within perinatal women engaging in opioid recovery services. Focus groups of these participants highlighted the importance of PSSs, reporting these individuals had a strong and positive impact on abstinence-based recovery. In contrast to the traditional role of professionals in such treatment settings, mentors often appeared to fulfil the role of a friend or advocate. While some have concerns over the potential blurring of boundaries [6, 11], for many, the positive impact of having a non-drug using friend is apparent. In their randomised trial, Litt et al. [19] reported that having one non-drinking friend present in a social network equated to a 27% increase in the likelihood of reporting abstinence in over 90% of Days at follow up. The longevity of this impact was also notable, with effects lasting up to 15months. A similar reduction in drug use was reported by Tracy et al. [37], with significantly greater reductions reported in individuals paired with a peer mentor compared to a treatment as usual group (*p* = 0.013), and this lasting over a 24 week period. While these findings show having non-drug using friends is not essential for recovery, this may be beneficial for some, and can be provided through LEROs.

### Aims of the study

While we have some evidence of the positive impact of social support within abstinence-based recovery from substance use disorder, scepticism remains on the integration of those with lived experience into healthcare [11]. Additionally, the amount of formal research specifically examining LEROs is limited. The current study aimed to gain insight into the impact of an established LERO for abstinence-based substance use recovery which focusses on social support and skills development. This study aimed to further inform policy and recommendations around the broader topic of social treatment within substance use disorder, as well as the narrower area of LEROs being used as a method of such treatment.

## Methods

### Intervention

Getting Clean are a volunteering project and not-for-profit organisation based in West Yorkshire, UK, formed by individuals who are in abstinence-based recovery from substance use disorder to provide social support and to other individuals working towards their own abstinence-based recovery. Within this LERO, the umbrella term of substance use refers to both drug and alcohol consumption. The organisation aims to; socially support people in drug and alcohol use recovery, to create a cleaner, more environmentally friendly local community and promote sustainability, and to provide participants with pathways to employment through practical skills workshops. LERO meetings are held consistently on a Friday afternoon, then complemented with other additional sessions during the week for social activities. All sessions are optional and typically combine peer-led support with social activities. While NF was familiar with this LERO though her community outreach work, both members of the research teams have no conflicts of interest with this charity and received no funding for this project.

### Participants

Guest, Bunce and Johnson [14] provide a general recommendation of gaining 12 participants for data saturation in interviews. Although, this threshold is dependent on a sample’s heterogeneity in life experiences and the depth of interviews, with others proposing 17 as a baseline number of interviews [13]. The current study aimed to recruit a minimum of 12 participants and then assess data saturation after each weekly session of interviews. After 15 interviews had been conducted both researchers agreed that data saturation had been reached.

Participants were required to be over 18 years old and a member of this LERO, although there was no minimum duration of involvement set. To be involved in this LERO participants must have the goal of abstinence from drug use. Members should not attend sessions when under the influence of substances. For the purpose of interviews, participants must also feel comfortable sharing information with the research team and have a sound level of verbal English language skills – both outlined in the consent form. Recruitment was conducted via convenience sampling and no reimbursement was provided for participating in the current study.

### Procedure

Ethical approval for this study was obtained from University of Bradford’s Research Ethics Panel, reference number E1179. The current study was introduced by the research team at a weekly LERO meeting. Participants were informed that this study aimed to examine their experiences using a LERO and introduced to the research team as university lecturers and researchers. Information was discussed over this three-hour period with questions asked and information sheets provided. No interviews were conducted in this first week to simply build rapport with participants and show genuine investment, adopting recommendations of trauma-informed research practices [17] – a crucial consideration in the topic of addiction [24]. This practice also allowed for participants to reflect on their involvement in the study and provide informed consent following this time period.

From the second week, semi-structured interviews were conducted with members who provided informed consent for participation. The current study was reintroduced at the beginning of sessions for continuous recruitment. Audio recorded interviews were conducted over a two-week period in private rooms on the site of meetings between the two members of the research team. Only the participant and one researcher were present during interviews. There were no restrictions on the duration of interviews, with this ranging from 19 to 49 min. Participants were reminded of the flexibility of these interviews; made aware they could skip any questions, pause for any breaks, or remove themselves from the study without any questions asked.

Semi-structured interviews began by collecting demographic data of gender, age, and duration of involvement in this organisation. Following this, participants were asked the question “How has being involved in this LERO impacted you?”. The research team – both trained and experienced in interviewing participants – then asked non-leading, open questions to prompt users to expand their points. Interviewers focussed on discussing the categories of; social, emotional, behavioural, and addiction-specific impacts. These headings were formed from the Biopsychosocial model and treatment [2, 10, 23] alongside specific addiction-based outcomes. Field notes were not kept during interviews to ensure participants’ comfort,adopting a trauma-informed research approach to this sensitive topic [17]. Therefore, a reflective notes were recorded immediately following each interview.

Data was anonymised upon recording with participants numbered. Files were transferred to a password protected, private computer which only team members could access and deleted from recording devices. Otter AI was originally used to transcribe audio files after reported accuracy in voice recognition [20, 29]. Despite these judgements, there were a high level of inaccuracies in these transcriptions – suspected due to a colloquial Northern dialect – meaning these interviews were instead manually transcribed by one of the researchers (GH). Transcripts were not returned to participants for comment or correction, although all participants were provided with researcher contact details incase any queries arose. Researchers were not contacted after these interviews.

### Analysis

Transcriptions of interviews were qualitatively analysed via thematic analysis, a method that allows themes to be derived from individual data to bring a collective meaning [16]. Analysis was completed using NVivo (version 15). To ensure all content is encapsulated from this dataset, an inductive approach was adopted. Braun and Clarke’s [5] stages of thematic analysis were completed,researchers first familiarised themselves with data, generated initial codes and then searched for themes. These first three phases were completed independently by both researchers before researchers reviewed their proposed themes, defined and named themes, and produced this report collaboratively. Participants were not sent the paper to provide feedback on the findings, although all were provided with researcher contact details if they wished to discuss anything following interviews. All selected quotes from a theme were written into a data extraction table (see supplementary material 1), with those most relevant discussed in the main body of this paper. A COREQ checklist was used to ensure transparent reporting of this study see supplementary material 2).

### Reflexivity

As two researchers involved in substance use recovery recovery work – one with over two decades of experience as a registered mental health nurse – we were mindful of how both our professional and personal experiences could shape our interpretations of data. Both GH and NF are white female lecturers working at UK universities. While we aimed to minimise power dynamics by attending meetings informally and not discussing our professional roles, these dynamics may still have influences the information participants shared, and in turn, the findings of this paper.

## Results

### Participants

15 participants (8 male, 7 female) had a mean age of 39.27 years (SD = 7.41). Ages ranged from 26 to 51 years. Time spent at Getting Clean ranged from two years – when the group had begun – to this being their first session. All participants listed multiple forms of previous substance use, with some also listing behavioural addictions (e.g. sexual relations and gambling). All participants approached consented to take part in the current study, and no participants withdrew from the study.

### Themes

**Fig. 1:**
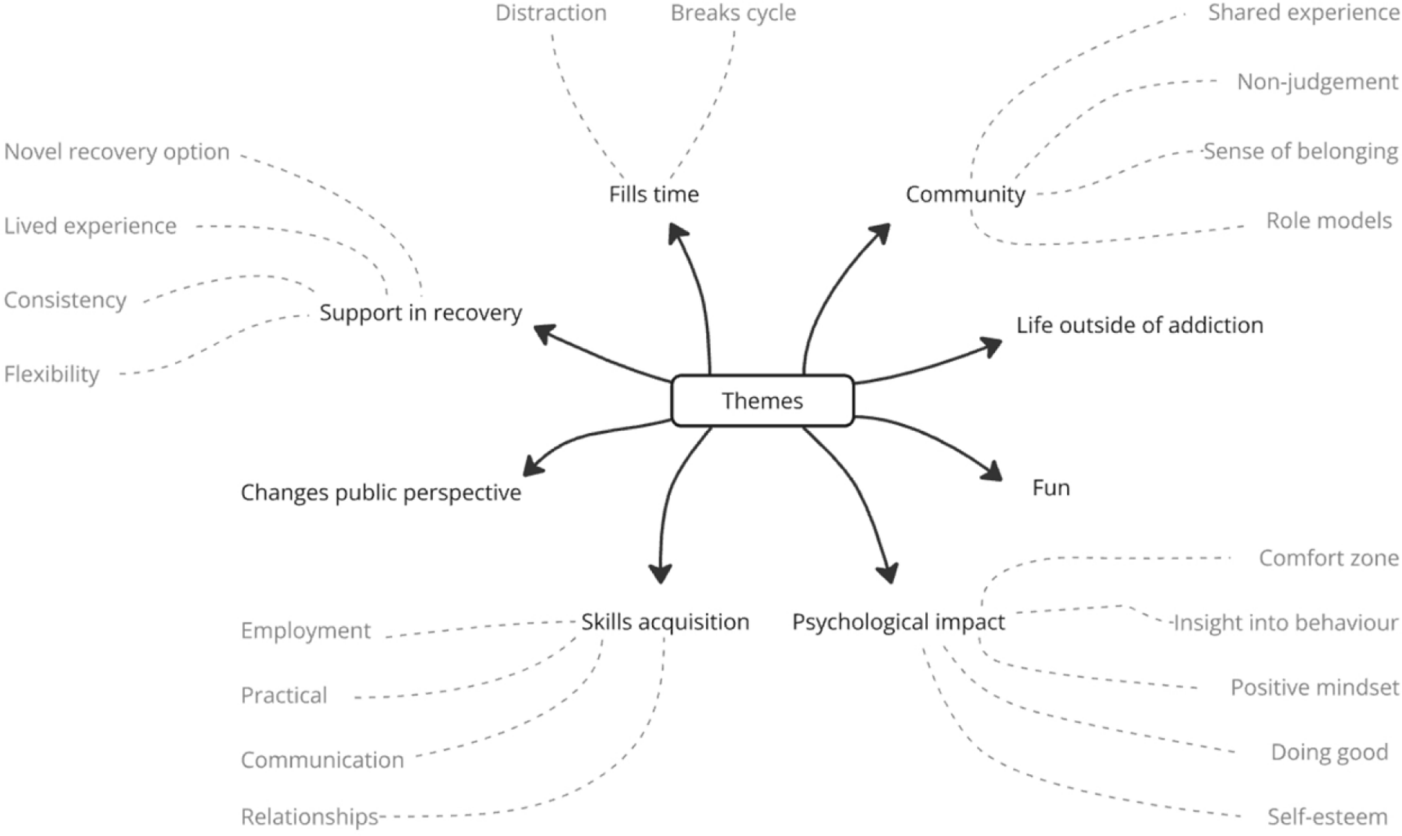
Mapped themes and sub-themes from data

**Fig. 2:**
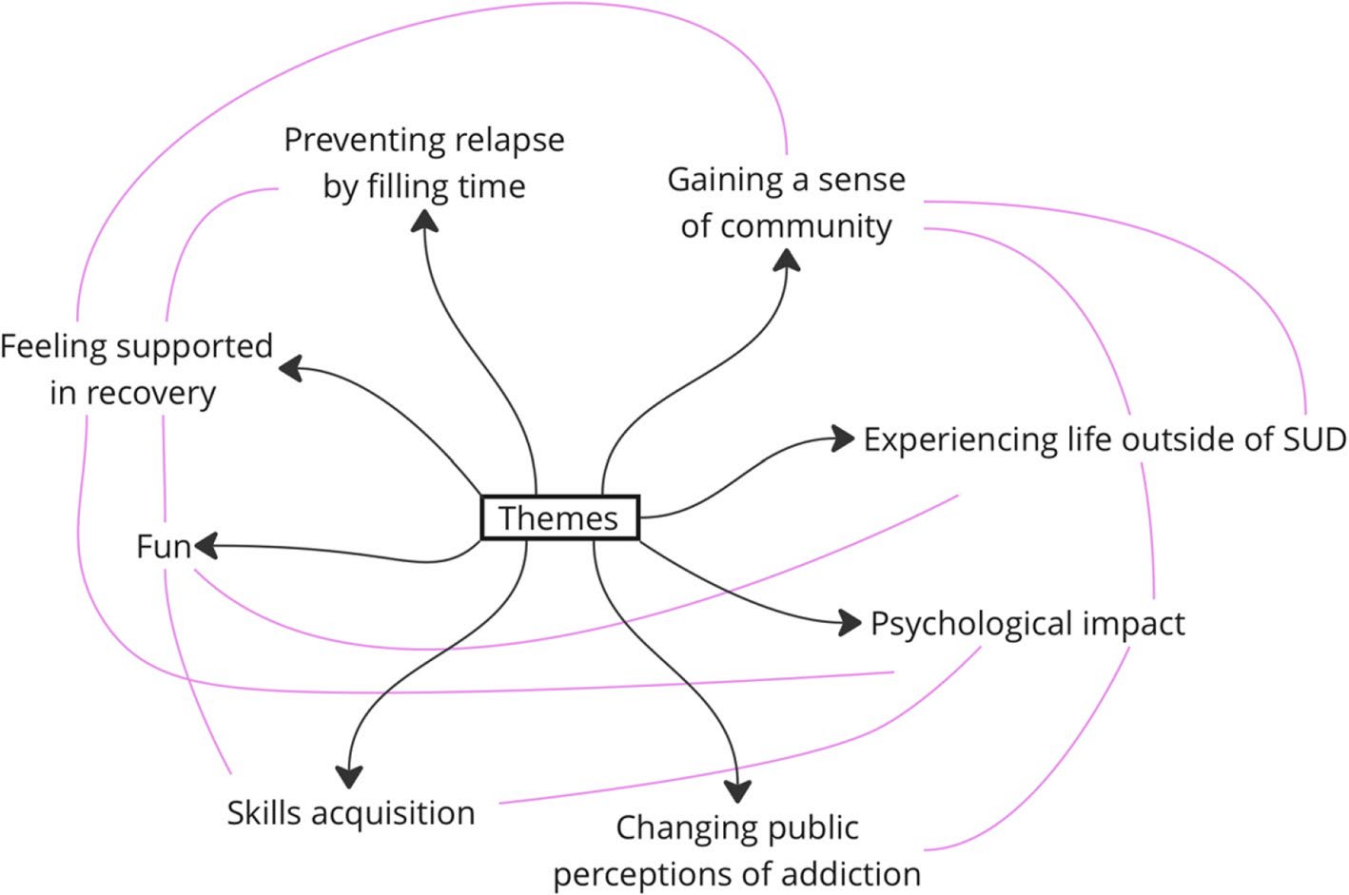
Interactions between themes

Eight themes were identified from interviews as impacts of this group. These were titled; Gaining a sense of community, Experiencing life outside of substance use dependency, Changes public perceptions of addiction, Psychological impact, Skills acquisition, Feeling supported in recovery, Fun, and Fills time. Four themes were standalone, whereas four had sub-themes. Themes and sub-themes are mapped in Fig. 1. Interactions between these themes are mapped in Fig. 2.

#### Feeling supported in recovery

Feeling supported in recovery was a theme somewhat expected to be present given this organisation’ aim, but participants emphasised its depth and sincerity compared to clinician treatment. Participants reported that they *‘feel truly supported here’* (P6, female) with this organisation being *‘here to listen to you, here to help you if [it]* can’ (P13, female). Support *‘tends to be recovery focussed, but it can be anything’* (P2, male) *‘at any time’* (P14, female) and is reciprocated by others with this lived experience of recovery. Many participants stressed the importance of feeling this genuine support, deeming it crucial for directly preventing relapses; *‘I don’t know what do without it, or I do…’* (P6, female), and reported this support as the reason people remain in this LERO with Participant 2 stating they *‘came for the activities first but [they] stay because of the support’* (P2, male).

This LERO format was recognised as *‘a different way of doing recovery’* to traditional clinical treatment, which felt more appropriate and supportive to meeting individuals’ needs than a classroom setting; *‘95% of addicts, they don't do classroom settings… whereas this is more informal, this is more hands on, you're relaxed, you can talk to each other’* (P2, male). Differences in rules in support were also mentioned; *‘You'd be welcomed back and supported from day one again, whereas if you're in rehab you're now homeless and probably going to use more because that's pretty fucking scary, isn't it?’* (P10, female) which potentially created a feeling of greater genuine support rather than being *‘all about numbers’* or *‘a box ticking exercise’* (P14, female). While we understand the reasons these rules are enforced in residential programmes, Participant 10 emphasised the long-term support provided at this LERO and the consequences when this is absent. Within this theme there were two sub-themes; Flexibility and trust placed in service users, and Dependable and consistent sessions.

Engagement in the programme was participant-led, with flexibility and trust placed in service users. Flexibility was reported in the level of involvement a service user has here, allowing people to come *‘as much or as little as you want’* (P14, female) which in turn allows people to have a life outside of recovery, for example *‘it still allows me to be a mum’* (P9, female). Flexibility also referred to participation in sessions which catered to one’s needs, with participant 8 reporting this LERO as *‘very easy going and I need that, easy going and okay with people saying no’* (P8, male). This relaxed and adaptable view may be overlooked, with participant 2 saying it *‘sounds basic, but it’s not. It means a lot and it feels respectful and truly caring’* (P2, male) highlighting the power of this group promoting autonomy in involvement and emphasising person-centred care.

The importance of having dependable and consistent sessions was highlighted within this data. When participants shared that they were unable to commit to sessions, they were greeted with reassurance on the consistency of this support remaining, such as *‘listen, that’s alright… just come back when you can and when you’re ready and nothing will have changed’* (P2, male). This consistency reflected the *‘patience for everyone’* here and *‘no end date’* on the opportunity for engagement (P2, male). Participants reported a door always being open for them here regardless of their progress, at *‘this place, always here, come whenever, no judgement’* (P10, female), particularly when participants experienced lapses where *‘this place is a constant… if you've been kicked out of your programme, you're still welcome here’* (P10, female), with *‘the support…always there for you’* (P11, male). Additionally, this group seemed to be perceived as a full community rather than an individual, with participant 14 saying *‘you know you can ring anyone here at any time and we’re always there for each other’* (P14, female). The physical consistency of meetings were practical for common knowledge of sessions; *‘it's every week at the same time and in the same place so even if you haven't got a phone, you haven't got anywhere to live’* (P10, female) individuals still knew this group was open to them due to its fixed occurrence pattern. Participant 10 added to this, branding these sessions as *‘always here… always a safe place you can go every Friday’* (P10, female).

#### Community

Alongside support during recovery, this LERO allowed service users to gain a sense of community. This community provided a *‘safe environment to be around people’* (P12, female) allowing individuals to *‘[get] away from those four walls… to a place of belonging, to see old friends, to meet new people’* (P11, male). Importantly, many were previously missing this community; *‘in active addiction I've been alone, I don't leave the house, I don't do anything, I hide away. And this was something to do as a group, a full group’* (P10, female). Participant 10 added to this, sharing they *‘have grown up with people around them who don't give a shit about them. But here, the level of care is just above and beyond’* (P10). Similarly, Participant 1 reported they *‘hadn't had this closeness since prison’* and that they *‘don’t know what [they'd] do without [it]’* (P1, male). This community seemed to blur the lines between *‘a full time friend but also a professional voice when you need it’ which was described as ‘special compared to other things on offer’* (P1, male) – something unique to this service which made individuals feel supported during recovery. Here, group members felt like *‘a big loving family that all want the best for each other’* (P15, female) where individuals are *‘made to feel welcome’* and *‘shown love’* (P15, female). Within this theme there were two sub-themes; feeling non-judgement due to shared SUD (substance use disorder) experience, and Interaction with relatable role models.

Feeling non-judgement due shared SUD experience was reported. Members of this community all have the shared experience of substance use disorder, meaning individuals are *‘around likeminded people’* (P15, female) who are all *‘overcoming the same things you're facing’* (P10, female). This shared understanding allowed deeper connections to form; talking to somebody *‘who gets exactly what you're saying’* (P10, female) means *‘there's so much stuff that just doesn't need to be said, there's so much stuff that we just know, that we don't have to explain to each other’* (P6, female). In turn, this allowed for more meaningful conversation, with one individual saying *‘She gave me the exact advice I needed. It was like she understood me better than I did because she's on the other side of it’* (P10, female). This shared experience primarily refers to experience of addiction but also covers shared life experiences. Individuals reported feeling represented in this community, with most people *‘from vulnerable backgrounds’* having faced things like *‘mental health, disability, [or] trauma’* (P11, male). Another participant noted social class as a shared experience, meeting people here who have *‘been in the same kind of background as me, the same kind of upbringing, council estates, homes’* (P1, male). Ultimately, this shared experience may facilitate a sense of belonging, with individuals reporting feeling isolated until meeting others facing these similar struggles; *‘I always thought I was alone, I always thought I was alone, that I felt different, that nobody has felt the same way I had or been to the same place I'd been’* (P15, female).

Due to their shared lived experiences of substance use disorder, participants felt this LERO was a safe space to share their feelings; *‘No matter what situation you're going through, nobody judges you… even if you relapse and come back, nobody judges you for it’* (P13, female). This was echoed by Participant 10 *‘you know you'd be welcomed back and supported from day one again’* (P10, female), highlighting how this non-judgement links to participants feeling supported in recovery. Perceived non-judgement facilitated honest and frank conversations between members, with *‘stuff that [individuals] wouldn't dare say to someone who hadn't been in addiction’ as ‘it would absolutely shake them’* (P6, female). This non-judgement was linked to the common lived experience here, with individuals feeling *‘more comfortable with [others] if they've experienced addiction because they get it more in my mind … it makes me feel a bit less judged’* (P1, male). ‘*I know some people are amazing and haven't experienced addiction, but I think most people, or maybe I'm just more comfortable with them if they've experienced addiction because they get it more in my mind … it makes me feel a bit less judged’*. Others also reported *‘feel more comfortable sharing things’* and therefore being *‘more open’* because at a LERO they’re with *‘people that get it’* (P4, male). Interestingly, some felt the area of the lived experience did not have to be within substance use to feel understood, and more so in the general topic of mental health, with one participant saying *‘you mentioned a past of eating disorders when saying hi and from that I was like oh she gets that nagging in the brain’* (P1, male) following a researcher’s discussion of their past eating disorders. As well as feeling truly understood, this lived experience had the positive of being *‘fully inspirational’* in a way that *‘you don't get that from counselling or talking through things with someone that hasn't been in addiction themselves’* (P1, male). Alongside feeling judgement, some participants questioned whether those without lived experience could truly understand addiction. A powerful quote from Participant 10 labelled addiction as impossible to understand by anyone, including themselves; *‘I will do it in hospital when my legs hanging off and I'm told I'm gonna die. I won't stop because I can't stop. How can anyone understand that? I don't understand that. That's what happens to me when I'm using and I don't understand it’* (P10, female), suggesting alongside perceived judgement there was optimism about feeling truly understood.

Crucially, this community of people with lived experience of substance use disorder allowed for interaction with relatable role models, with *‘so much inspiration here’* (P1, male) and *‘a sense of hope*’ (P5, female) from people *‘in front of me, doing everything I’m trying to do’* (P14, female). This ultimately proved to service users that recovery is possible; *‘You can't not believe it's possible when people in front of you are doing it. They're recovering so, so why can't you?’* (P1, male)*. Importantly, this ‘feels like realistic but still inspirational’* (P4, male), perhaps because individuals are *‘around other people who have been where [they’ve] been and have come through it’* (P5, female). This recovery role modelling *‘soothes [one’s] worries’* and *‘those questions on loop about the future’* (P2, male) by showing that *‘if you just continue doing this, coming here, turning up, that's enough. You can do it’* (P10, female) making sustained abstinence feel more realistic and achievable. Additionally, the authenticity of these role models in normalising that change is difficult provides reassurance and inspiration; *‘I just think there's other people here dealing with other things and still managing to do it, why can't I just follow what they're doing?’* (P15, male). As well as role models in terms of recovery, individuals are *‘so vocal about struggles and how they're doing’*, sharing similar feelings to others, can provide *‘the strength and hope to carry on’* (P10, female).

#### Psychological impact

Getting Clean had psychological impact on participants in the current study. Generally making people *‘feel good about [themselves]’, ‘listened to and heard’* (P2, male), feelings that particularly *‘[matter] when [individuals] might not have had that before’* (P10, female). These groups allow people to feel *‘normal, like a member of society doing things [they] enjoy’* (P4, male). Within this theme there were three sub-themes; Improved self-worth, Adopting a rational and positive mindset, and Feeling a sense of belonging.

The most common psychological impact here was improved self-worth, with individuals *‘having their eyes opened here in terms of what they can actually like do’* which *‘goes a long way’* (P1, male). From interacting here, participants *‘feel busy and useful’* and are reminded that they have strengths, such as being *‘a good team player’* (P11, male). These groups have *‘helped bring confidence back’* and *‘helped [individuals] learn who [they are]’* (P11, male). One participant spoke of being involved in the current study, listing this as an example of something they wouldn’t previous participate in, but have done due to raised self-worth; *‘I don't think I would've spoken to anybody then because my self-esteem and how I treated myself was so low I would have presumed that even though you came here today and explained what you were doing, I would presume, no she doesn't mean me, nobody wants to hear from me’* (P1, male). Building this self-worth was reported as crucial for those specifically facing substance use recovery, because often *‘there's a lot of people who don't realise the skills that they have because it's been, it's been lost in a mire of years of hurt and abuse’*, with many feeling *‘worthless’* (P1, male). Some people have *‘nothing’* (P6, female) when they come here, participant 6 said, with *‘no job experience, no skills, no knowledge of how to behave’* and for these people, self-esteem must be focussed on for individuals *‘to feel useful—that's so important’* (P6, female).

Participants also reported being involved in making soap and donating them which *‘makes [them] feel amazing’* (P1, male) because they’re *‘doing sommat for others’* (P2, male) and *‘giving a bit back to the community’* (P2, male). Participant 15 echoed this, noting that this involvement in activities is important because it makes them *‘feel good inside’*, describing this reward as *‘a better feeling than any substances I've ever taken’* (P15, male). This improved self-worth and one’s perceived value, with these acts proving *‘even though [an individual has] relapsed [they] can still help people’* (P12, female) and being *‘something you can look at and feel proud of being a part of’* (P4, male). The importance of developing self-worth was highlighted by participant 1, who stated it needed *‘building up … because you just don't think you've got a fucking worth to life’* (P1, male) in addiction.

Participants also reported that these groups helped them in adopting a rational and positive mindset being adopted. *‘Here they're good at focussing on the now and doing what you can do now’* participant 1 said (P1, male). They reported they were now *‘able to say look this happened, and I can't change that, but I can show you these things I've been doing now that I'm dead proud of’* (P1, male), showing acceptance. Others also reported this realistic acceptance, saying they’d *‘done a lot of bad things, but that’s past tense’* (P4, male), clarifying that they were *‘not like proud of [their] past, but just accepting’* it and thinking *‘but look what we’re doing now’* (P10, female). This positive mindset, especially in relation to accepting their past, was listed as a reason for not relapsing, with these weekly meeting lifting mood and confidence in oneself; *‘Just having that hope and this uplift, sometimes that's kept me from relapsing’* (P10, female).

Gaining a sense of community also had the psychological impact of providing a sense of belonging to these service users, establishing *‘a safe space’* (P1, male) where individuals are able *‘to feel a part of something, made to feel valued, part of a team, a family’* – things they’d *‘never had’* (P15, male). This was typically lacking for participants when they were in active addiction, whereas individuals have *‘now realised that [they are] a part here, [they’re] not invisible now because many people feel completely invisible, unseen, like nobody knows anything about them’* (P4, male). Others echoed this, with that sense of belonging making them *‘feel normal’* because *‘you don't have a group to have a laugh within active addiction, you know, you're isolated and your routine revolves around the dealer, but here you get a taste of what life should be like with that fun and support’* (P10, female). This sense of belonging allowed for individuals to be authentic, meaning they *‘don’t have to hide behind a mask’* (P13, female) yet still have *‘somewhere that [they] belong, where [they] fit in’* (P15, male); making them *‘feel wanted’* (P13, female).

#### Skills acquisition

Within these groups, skills acquisition occurred for participants who are now *‘achieving things that six months ago [they were] convinced [they would] never be able to do’* (P9, female). Some of these skills were intentionally developed through planned activities, e.g. soap making, whereas other skills came naturally over time through attending these sessions. These skills gained were divided into three sub-themes; Social and emotional skills, Practical skills, and Insight into behaviour.

Social and emotional skills were built naturally in sessions because *‘everyone talks here’* (P1, male). This has *‘changed [individuals’] social boundaries and skills’* (P1, male) meaning participants are making huge amounts of personal progress; *‘I don't think I would've spoken to anybody then’* (P1, male) including being involved in this study. These skills are particularly important to build up as many report losing them during their substance use dependency and associated circumstances; *‘When I came into recovery I came straight out of prison so I didn’t know how to socialise you know, I'd lost all of them skills, and Getting Clean has enabled me to get them back’* (P7, male). Participant 15 additionally mentioned these skills needing to be relearned; *‘When I first came I had to fight a few fears, I wanted to bolt. I thought bloody hell what are these people doing, they're all saying 'oh your alright?' 'nice to see you'… I was thinking 'what do they want?’* (P15, male). Learning these communication skills seem to have led to improved self-confidence; *‘The first thing I said to them was 'can you put me in the storeroom because I'm not a people person?' … I'll speak to anyone now!’* (P14, female). Participant 14 also reported they have now *‘got a voice’* – *‘[they] didn't used to have one, but [they’ve] got one now’* (P14, female). Building these skills has also assisted recovery directly, preventing relapses by encouraging participants not to *‘bottle it up and get overwhelmed’* (P15, male), instead being open about their feelings. Through participation in these sessions, agency and autonomy improved, as participants report they have also gained *‘social boundaries’* (P1, male). Participant 14 reported that they *‘used to do things against [their] will, but now if [they] don't want to do something, [they] just say no'* (P14, female). From these sessions, they have *‘learned that no is a full sentence, you don't need to explain why, it's just no’* (P14, female). In turn, these have impacted others outside of this immediate community too, with participants reporting their *‘relationship with [their] family [have] massively improved since doing this’* (P1, male) and that their parents *‘love that [they’re] here’* (P10, female). Sessions are also facilitating participants to learn *‘how to be a mum now’* (P14, female) by being *‘more understanding’* (P9, female) and *‘role modelling the things [their children are] struggling to deal with’* (P9, female).

Practical skills have come from the activities hosted within weekly sessions, with individuals demonstrating their own capabilities to themselves; *‘I can cook, I can make this, I can give something back, I can do a job and I can complete a task’* (P6, female). This has different levels of significance for participants depending on their backgrounds, but *‘a lot of people haven't had that before so it's definitely huge’* (P6, female). These practical skills have an employment focus, by allowing participants the opportunity *‘learning skills here that [they] can transfer into life and transfer into jobs in the future’* (P3, male). By being involved in this organisation, this means participants *‘have stuff to put on [their] CV’* (P1, male) and *‘can put [founders] down as a reference!’* (P7, male). This filled a perceived issue and worry for some who *‘have that CV stress’,* questioning *‘what do I say about this time off?’* (P9, female) where *‘there's just a big gap on there, like a massive black hole on paper’* (P1, male). Participants reported this employment focus as *‘something [they’re] excited about’* because they are *‘so keen to work again’* (P5, female).

Participants have also gained insight into behaviour from these groups, with some now *‘better at thinking about [their] emotions and like breaking things down in my head’* (P4, male) and understanding that *‘[their] brain has weird ways to do things’* meaning they *‘have to break down and work out and question’* (P1, male) their feelings before acting upon them. Participants have reported being *‘very self-aware now’* (P14, female), seeing struggle as inevitable and something that is simply *‘gonna happen’* (P12, female), but understanding that drug use *‘probably would bring a bit of relief but literally for about ten seconds and then [their thoughts would] come pounding back’* (P15, male). Another participant also reported being able to apply this learned insight to others like their daughter, saying *‘I do feel like I've gained more understanding in her mental health’* (P9, female).

#### Life outside of substance use disorder

Participants reported being connected to life outside of substance use disorder through this intervention. Some learned about new interests they gained such as climate change which feels *‘refreshing and cool’* (P2, male) to be able to focus on over the *‘bigger fish to fry when [they’re] stuck trying to score’* (P2, male). These new interests meant people have more of *‘a purpose’* (P14, female) outside of meetings and also gained *‘some sort of, yeah, identity… [this LERO] has helped me to find that’* (P11, male). Others mentioned they reconnect with old interests and were *‘slowly finding [themselves] again’* (P13, female) and *‘feel like [they are] being the real genuine me, the person [they were] before addiction’* (P15, male). Connecting with old interests *‘[brought] back memories, happy memories’* (P7, male) and *‘the bits [they] want to remember and the childhood members you love’* (P7, male). Others mentioned the future life they are building outside of addiction, naming sessions as *‘a way to be a productive member of society’* (P3, male) and *‘an opportunity of practising taking [their] recovery into other aspects of life, so that's like socialising, meeting new people, looking at employment’* (P9, female). These acts *‘[help] with a fulfilling life’* (P9, female) and also get individuals *‘ready for the outside world’* (P14, female) if they are currently in a treatment facility. Participant 10 highlighted the importance of connecting with this life outside of addiction, with it making them *‘feel normal’* and *‘get a taste of what life should be’* (P10, female). And for some, this new life was *‘a life beyond [their] wildest dreams from all those years in addiction’* (P15, male). This was a standalone theme with no themes.

#### Fun

Having fun was a prominent experience for participants. Participants reported that their days here have ‘felt fun and silly’, which mattered hugely as this is *‘what [they] were missing’* (P2, male) within their recovery. Another participant reported this as feeling *‘refreshing’* and *‘a bit childlike but maybe in a good way’* (P8, male) linking this theme to making a life outside of substance use disorder and reconnecting with old hobbies. This fun provided *‘a bit of escapism’* for some, *‘to get away from that (recovery) cos that’s too much’* (P12, female) with many seeing this as a part of recovery, but also a break from traditional sessions. *‘Here is more of a laugh’* participant 3 said, adding that they *‘actually enjoy it’* when comparing it to traditional services which *‘can all be quite serious’* (P6, female). Instead, these sessions felt *‘different to what [participants are] doing day in day out’* (P6, female) and provided an environment that was *‘more relaxed and a bit more informal’* (P7, male). Having fun impacted participants by *‘making them feel normal’*; something missing because they *‘don’t have a group to have a laugh within active addiction, [they’re] isolated and [their] routine revolves around the dealer’* (P10, female). While this may seem like *‘just having a laugh and a joke’* participants can feel immense pressure to stay abstinent within recovery; they *‘can come in this room and feel the weight of the world on [their] shoulders’* (P10, female). Here, *‘that fun stuff, and just having that hope and this uplift, sometimes that’s kept me from relapsing’* participant 10 reported, highlighting that these groups can directly prevent relapse. Others echoed this, saying they aim just to get to these sessions knowing that *‘by the time [they] leave [they’ll] feel better’* (P12, female). Ultimately, this fun gave individuals *‘a taste of what life should be like with that fun and support’* (P10, female), which could in turn remove a reason for participants to relapse. This was a standalone theme with no themes.

#### Preventing relapse by filling time

Participants reported having a lot of time on their hands, typically due to being in a daytime facility and not working, with this LERO acting as something which prevented relapse by filling time. These groups are *‘something extra to do during the day’* (P7, male) which seemed important to many individuals as this is their normality; *‘I need to keep busy because I was busy before and during a lot of my drug use’* (P2, male). If participants were in traditional treatment, their days were structured, although meetings *‘only last like an hour, an hour and a half, so this gives [them] other things to do’* (P14, female). One participant had recently been required to leave a traditional live-in centre, questioning *‘what am I gonna do with all my time now I'm not doing group sessions?’* and highlighting the difference in rules here in this treatment option. Furthermore, participant 6 compared these to *‘youth clubs’* to provide that fun, but also to prevent people from turning to negative behaviours out of boredom. The importance of filling time was apparent, with participant 1 stating *‘when I've got time on my hands that's a problem, that's when problems start.’* (P1, male). Instead, coming to sessions allowed users to *‘occupy [their] time so [they weren’t] sat there in [their] own bed thinking about using.’* (P1, male). Concerns over free time were particularly strong about weekend. Typically, traditional services run from Monday to Friday meaning *‘It's the weekends that are lots of time for [yourself]’* (P4, male). By filling weekend slots, these groups may have prevented many from relapsing; *‘I think I'd have been closer to relapsing if I didn't fill my weekends’* (P3, male). This can occur through breaking the cycle users have in addiction. The cycle discussed referred to the routine of *‘get up, go see your dealer, and that's even if you've slept, go see your dealer and that's it, that's your only form of routine’* (P10, female). Instead, *‘this place is instilling a routine for people. That routine of giving people a place to go and something to do that doesn't involve that cycle’* (P10, female). Participants specifically focussed on the challenges they face at weekends again; with participant 3 noting the importance of groups *‘especially on the Fridays, some people struggle with Fridays’* (P3, male). Additionally, participant 10 listed these sessions as a way to directly prevent relapsing by breaking these weekend routines; *‘On some Fridays I've been ready to relapse and here, from going here on a Friday, it's kept me from relapsing’* (P10, female).

These sessions also act as a distraction from thinking about using substances, by filling some of the time individuals have without traditional support; *‘It helps me with those thoughts you know … I finish group at 12 and then don’t go to a meeting till 6:30 so it's a long time to be with yourself’* (P13, female). This is important as users reported having *‘too much extra time’* and that their *‘mind runs… to that overwhelming feeling’* (P8, male). This seemed particularly useful for those at the beginning of their recovery when *‘it’s not good to be sat around with your head’* (P1, male) and when during *‘those couple of hours at home, [their] brain just, it just thinks about using’* (P13, female). Instead, Getting Clean provides respite; *‘a nice place to be’* but importantly, somewhere *‘not forget everything, but to take a break from it, from all your struggles, and get on with something that distracts you from that’* (P13, female) via the fun activities that individuals take part in. Participant 12 reported wanting to relapse the morning prior to their interview, but coming to sessions specifically to distract them, while also improve their mood; *‘This morning I thought to myself, do you know what, I just wanna go score, but I knew this was on and I phoned a friend who said she was coming and I though get yourself down here, even though I didn't really wanna, I just thought I'd get myself out and down here and it'll kill a few hours, you know? And by the time I leave, I'll feel better, I'll have had a laugh here, you know, they'll have lifted my spirits’* (P12, female).

#### Changing public perspective

A secondary effect of these groups were that they could be changing public perspective around addiction, prompting the wider community to *‘start looking deeper into it and seeing a person behind that’* substance use (P1, male). The perceived stigma these participants felt from those outside of addiction was evident. Participant 10 said *‘there's so much stigma around addiction but from this we're getting to speak to people and change their image of addiction’* (P10, female) with participant 13 adding that *‘people can look down on us’* (P13, female). Aims of this group for many were to *‘hopefully [give] people a different perspective on addiction and what recovery looks like’* (P7, male) and for the community to *‘hopefully see us for the good we're doing’* (P1, male). Participant 2 stated similar goal; *‘people can see, yeah, [they were] an addict but [they’re] looking to change my life and do good’* (P2, male). Furthermore, Participant 1 said *‘Where she saw you scoring at the bottom of the road she's now seeing you pick her rubbish up and that must be cool, right?’* (P1, male), highlighting the enthusiasm participants had at this opportunity to change attitudes. Additionally, this aim to change perspective was typically linked to one’s improved self-worth about their past; *‘I could tell you that I've been to prison but I'm a good person, but would you believe someone saying that? I don’t know, but I could show you the good I'm doing by cleaning up and going to markets and prove that’* (P1, male), perhaps aiming to change perspective to feel valued in the wider community.

## Discussion

### Findings

The current paper examined the experiences of 15 individuals who were attending a LERO to support their abstinence-based substance use disorder recovery via the completion of semi-structured interviews. Inductive thematic analysis allowed for a deep understanding to be gained into the experiences within this LERO, with eight emerged themes; this group primarily left individuals feeling supported in recovery by experiencing life outside of substance use disorder through having fun and focussing on skills acquisition. Coming to this organisation and participating in these activities prevented relapse by filling time around weekends when traditional services did not operate, but importantly, also led to members gaining a sense of community. This network had a positive psychological impact for these participants, with many reporting improved self-worth. Furthermore, the community and environmental focus of this LERO meant users could be involved in changing public perception around the possibility of recovery; reducing stigma around this topic.

### Existing literature

While this paper did not collect objective measures of abstinence rates, meaning it cannot add to Tracy and Wallace’s [36] systematic review, both this paper and the current study highlighted the coping skills and self-efficacy which can be improved through social support provision. Similarly the current findings highlighted improved quality of life through the social connections this LERO facilitated and real service user satisfaction from these participants, supporting the findings of Kulik and Shah [18] and Bathish [3]. This satisfaction is in contrast to self reported poor experiences in traditional, healthcare profession led, addiction services – strengthening the conclusions of Crapanzano [7] – with participants in the current study specifically reporting feelings of judgement and not being understood by these clinicians. Many themes in the current study pointed to Alexander’s [1] dislocation theory of addiction with factors such as isolation, detachment from society and a loss of identity may lead to substance dependence were mirrored in the current paper. Furthermore, the sense of belongingness and the inspiration of shared goals participants reported can highlight the role Social Identity Theory may plan in substance use disorder recovery [4].

These groups also built communication and relationship based skills, self-esteem, and a routine for these participants, further making them feel integrated into society and *‘get a taste of what life should be like’* (P10). These findings support the work of Pickard [28], with the thematic analysis similarly identifying that a focus on social integration and relationship formation appeared crucial for participants. Furthermore, participants often focussed on the importance of having likeminded people around them and having skills-based activities within these sessions. These themes can strengthen the concept that those recovering from addiction typically undergo an identity shift when changing their substance use – supporting the mindset of Best et al. [4] and Robertson and Nesvåg [31] who propose that further support is needed during this difficult to navigate transition for treatment success. Finally, this paper strengthened the claims of Dame Carol Black that ‘recovery is about more than just treatment’ (Black, 2020), with participants in the current study placing importance on the employment skills this organisation provided. Building a life outside of substance use disorder, having fun, and support participants with administrative tasks relating to housing and finances also made this LERO stand out in comparison to traditional services.

### Strengths and limitations

The current paper gave space to unheard voices from an often stigmatised community, having a valuable, positive impact on our understanding of first-hand perspectives of abstinence-based substance use disorder recovery. This small-scale study also helped to bridge a reported gap between service users, clinicians, and academics which must be continued in order to reduce stigma around substance use disorder, particularly in healthcare professionals, whilst also increasing our knowledge of the topic [32]. Furthermore, this study aimed to conduct this research in a trauma-informed manner – something we hope future research will take note of.

Although this paper holds great value, naturally there are limitations with all research. This study was conducted at a single site LERO meaning our findings may not be generalisable elsewhere. Despite this, data saturation was gained within these interviews and a vast range of themes were identified from the rich data provided by participants. Another potential limitation of the current study was that only active users of this LERO were recruited. This means that voices may have been missed in this data from individuals who opted out of their involvement with this organisation – data which could be valuable for providing further, and perhaps contrasting, insight to LEROs. Future research should aim include a wider range of participants from multiple LEROs, including individuals who are no longer active users of these organisations. Additionally, employing mixed methods approaches to examine behavioural outcomes alongside these experiences could provide deeper insights into the impact of LEROs within addiction recovery.

### Implications

The findings of this paper hold implications for healthcare recommendations. While no objective data was collected on abstinence and this sample was limited in scope, it is clear that this LERO played a crucial role in the abstinence-based recovery for these participants. Many participants emphasised the importance of social support within their recovery; a focus which seems to be lacking in the traditional treatment route currently provided through the NHS, therefore highlighting how a LERO could be used alongside clinical addiction treatment. When comparing this LERO to traditional treatment, participants commonly reported an absence of fun in these other programmes, despite the link between well-being, mental illness, and substance use disorder well known. Alongside existing literature, the current study supported the adoption of a holistic view of addiction recovery, focussing on continuing abstinence but also building social ties, hobbies, and a life outside of substance use disorder.

These findings also highlighted the demand for involving individuals with lived experience within healthcare services where possible, with some participants expressing frustration around clinicians delivering treatment without lived experience in this area. While this stance may demerit the skills of clinicians, identifying possible areas to incorporate those with lived experience of substance use disorder into healthcare may alleviate feelings of judgement and the ‘us versus them’ mentality that many currently perceived within clinical treatment. While further research and implementation planning is needed, this study could partially inform future service development.

## Conclusion

The current study examined the experiences of 15 members using a West Yorkshire-based LERO supporting abstinence-based substance use disorder recovery via semi-structured interviews. Through inductive thematic analysis authors identified eight themes; Gaining a sense of community, Experiencing life outside of substance use dependency, Changes public perceptions of addiction, Psychological impact, Skills acquisition, Feeling supported in recovery, Fun, and Fills time. While only a small number of voices at a single LERO were examined, this rich data clearly highlighted the benefits of this social support. Specifically, the importance of having those with lived experience, feeling valued and a part of a society, and making time for enjoyment were emphasised by participants. This paper evidences the benefits of using a LERO alongside clinical substance use disorder treatment and involving experts by experience where possible to help address ongoing issues around belongingness and stigma among service users.

## Supplementary Information

Supplementary Material 1.

Supplementary Material 2.

## Data Availability

Data cannot be shared in full to preserve anonymity for participants. This is crucial due to the sensitivity of topics discussed and low number of participants in the current study. However, a full data extraction table of relevant quotes can be accessed as a supplementary material.
