# Supplementary material for: Experiences of a lived experience recovery organisation for those in abstinence-based substance use recovery: a thematic analysis
Source: Subst Abuse Treat Prev Policy. 2025 Oct 9;20:41. doi: 10.1186/s13011-025-00671-9 (PMC12512285; doi:10.1186/s13011-025-00671-9)
Supplement: Supplementary file 1 — Supplementary Material 1. [file 13011_2025_671_MOESM1_ESM.docx]

Table 1. Quotes table from thematic analysis.

| Theme | Sub-theme | Quote (participant ID) |
| --- | --- | --- |
| Gaining a sense of community |  | ‘We all build each other up… they are good good people and I don’t know what I'd do without them’ (P1, male).  ‘I hadn't had this closeness since prison’ (P1, male).  ‘it's an arm round your shoulder, it's a random text, it's a full time friend but also a professional voice when you need it, and, and that's special compared to other things on offer’ (P1, male).  ‘I can go a bit quiet sometimes, and here I'm always asked if I'm okay and I don't think that would be noticed elsewhere. You know, someone else could think, oh nice, he's behaving’ (P2, male).  ‘They'll send me random texts sometimes like what they're grateful for. Things like that feel kind of silly, but remind you that that they actually care and it's a bit more on the friendship side of things when you compare it to other stuff out there’ (P3, male).  ‘Here though, its like, it literally feels like friends just meeting up, meeting up and doing good things’ (P3, male).  ‘I just felt like people cared. I came down and instantly people wanted to get to know me, like, I felt popular! Yeah people felt genuinely nice and genuinely interested in me. Yeah, the inclusivity of it all, that's really good’ (P4, male).  ‘It felt so open and warm here’ (P5, female).  ‘They're just supporting me and keeping me around people. You know, if I'm sat at home by myself I'm just not gonna be in a good place’ (P6, female).  ‘Growing up in school and stuff I struggled to socialise because I'm on the autistic spectrum, I struggled to engage with people, here everyone's so like welcoming and friendly. It's so much easier’ (P7, male).  ‘It puts you in touch with other people, you know, other people who get it, they get addiction and also inspire you’ (P10, female).  ‘They care here, they really do, and that's the most important part to me because people in addiction, generally they will have grown up with people around them who don't give a shit about them. But here, the level of care is just above and beyond’ (P10, female).  ‘When I've been in active addiction I've been alone, I don't leave the house, I don't do anything, I hide away. And this was something to do as a group, a full group’ (P10, female). ‘You're getting away from those four walls and to a place of belonging, to see old friends, to meet new people, you know, people who have suffered with the same things you're going through, so that's belonging, that's acceptance’ (P11, male).  ‘It's a safe environment to be around people’ (P12, female).  ‘We’re like a family here. They love you, they give you support. I feel like we could, well, not handle anything, but you know, you know they've got your back’ (P13, female).  ‘I came along and was made to feel welcome, shown love’ (P15, male).  ‘There was always ulterior motives there (traditional treatment), but here there's not, we're just a big loving family that all want the best for each other’ (P15, male). |
|  | Feeling non-judgement due to shared SUD experience | ‘I used to think he hasn't been where I have, but when I had the opportunity to see people I knew had similar experiences to me because, because, I had literally been doing it with them… I knew they got it’ (P1, male).  ‘From someone who has been in the same kind of background as me, the same kind of upbringing, council estates, homes’ (P1, male).  ‘And here, it's like people, people get it and don't judge, but also really do support you’ (P1, male). ‘That lived experience, going back to it, but it's so massively important to me. I know some people are amazing and haven't experienced addiction, but I think most people, or maybe I'm just more comfortable with them if they've experienced addiction because they get it more in my mind ... it makes me feel a bit less judged’ (P1, male).  ‘I would not engage in treatment if the person was like oh yeah, I’ve read this in a book. No disrespect, I just wouldn't do it, like fuck off you don't know what you're really talking about’ (P1, male).  ‘It's fully inspirational and you don't get that from counselling or talking through things with someone that hasn't been in addiction themselves’ (P1, male).  ‘That lived experience, going back to it, but it's so massively important to me. I know some people are amazing and haven't experienced addiction, but I think most people, or maybe I'm just more comfortable with them if they've experienced addiction because they get it more in my mind ... it makes me feel a bit less judged’ (P1, male).  ‘You can read as many books and look at as many papers and computers as you want, but it's not the same. Not in a rude way, it's just so so intense’ (P1, male).  ‘I know you mentioned a past of eating disorders when saying hi and from that I was like oh she gets that nagging in the brain that some people, some fucking lucky people, can't imagine’ (P2, male).  ‘The volunteers, they go through a hard time but they still turn up, it's always a commitment. I like that they don't pretend they're not going through a hard time’ (P3, male).  ‘The openness stands out here and a like positive stance, that yeah you've done a lot of bad things, but that's past tense’ (P4, male).  ‘There's always emotional support there, always, you've always got that and it's from people that get it… I think I feel more comfortable sharing things and it makes me feel more open, even though experts in quotes could be the same’ (P4, male).  ‘The knowledge granted, yeah that's fine, but for the deepness, the connection and truly the severity of thoughts and impulses. It think that's only ever gonna come from someone whose experienced some form of addiction’ (P4, male).  ‘When you go to a meeting there's so much stuff that just doesn't need to be said, there's so much stuff that we just know, that we don't have to explain to each other’ (P6, female).  ‘Most of us have been in drug services and had drug and alcohol workers who have not been in addiction and it's, it's a completely unique experience. We struggle to understand our own addiction so, so how is somebody else without that..?’ (P6, female).  ‘I think you can work with addicts if you're not an addict, but you cannot understand truly the experience of an addict, it's impossible to get that strength. Yeah it's impossible. How on earth can you say oh yeah I understand when I say, you know, I will continue injecting drugs, I will use dirty needles, I will do it in hospital when my legs hanging off and I'm told I'm gonna die. I won't stop because I can't stop. How can anyone understand that? I don't understand that. That's what happens to me when I'm using and I don't understand it’ (P6, female).  ‘There's stuff that I wouldn't dare say to someone who hadn't been in addiction because there's some experiences I've had that, and some things that I've done that I've got so much shame around, you know, that if I said that to a person who hadn't experienced addiction it would absolutely shake them’ (P6, female).  ‘I help other people in recovery as well who aren't quite as far in as me’ (P9, female).  ‘There's no judgement - people do have really deep conversations over a brew’ (P9, female).  ‘They're overcoming the same things you're facing… they get addiction and also inspire you’ (P10, female).  ‘She gave me the exact advice I needed. It was like she understood me better than I did because she's on the other side of it’ (P10, female).  ‘You chat with someone else who, who gets exactly what you're saying and they've managed to overcome it so you believe you can too’ (P10, female).  ‘Once you've had this, its hard to go back to using again, but if you did, you don't have fear and shame over it, you know you'd be welcomed back and supported from day one again’ (P10, female).  ‘Most people here are individuals from vulnerable backgrounds, it's addiction, whether it's mental health, disability, trauma’ (P11, male).  ‘There's nobody there discriminating anyone else, there's nobody taking the mick out of another person’ (P11, male).  ‘I'm mixing with people who are all doing the same thing’ (P12, female).  ‘You're not judged and you can just share where you're at’ (P12, female).  ‘You can talk to people about what's going on with you, and they've all been through similar things so you know, you can like, so, you can hear their thoughts and get a different perspective on something’ (P12, female).  ‘No matter what situation you're going through, nobody judges you’ (P13, female).  ‘Even if you relapse and come back, nobody, nobody judges you for it, nobody will say like you shouldn't have done that, they just welcome you back’ (P13, female).  ‘It got to the point where they'd come in and I'd say have you got any experience in this? Have you ever taken drugs? And they'd say no no, and I'd say it's not gonna work then. How can you tell me to get off a script and put things in place if you've never done it?’ (P14, female).  ‘Being around likeminded people, do you know what I mean? Cos I always thought I was alone, I always thought I was alone, that I felt different, that nobody has felt the same way I had or been to the same place I'd been’ (P15, male).  ‘Being around those people with that life experience, who has used drugs similar to me or who had similar life experiences, like hearing it from them, do you know what I mean, it hits differently’ (P15, male).  ‘I'm not saying they genuinely don't want to help, but it felt like, I felt like they never truly understood what was going on’ (P15, male). |
|  | Interaction with relatable role models | ‘He has been a massive massive inspiration to me’ (P1, male).  ‘There's so much inspiration here’ (P1, male).  ‘It's fully inspirational and you don't get that from counselling or talking through things with someone that hasn't been in addiction themselves’ (P1, male).  ‘Role models are here. You can't not believe it's possible when people in front of you are doing it. They're recovering so, so why can't you?’ (P1, male).  ‘Seeing and knowing people steps ahead of you in recovery helps soothes my worries, that, those questions on loop about the future’ (P2, male).  ‘It feels like realistic but still inspirational’ (P4, male).  ‘When you're around other people who have been where you've been and have come through it and are a lot further than you, it gives you a sense of hope’ (P5, female).  ‘It's about being around clean people and hearing from them, hearing the clean time they've got and how they're overcoming the same things you're facing’ (P10, female).  ‘It's that, that success that gives people hope, you know, if they're doing it I can do it too. If you just continue doing this, coming here, turning up, that's enough. You can do it’ (P10, female).  ‘The people here, they're so vocal about struggles and how they're doing and even that, that courage to voice what usually I'm feeling, to do that for me without realising, that courage gives me the strength and hope to carry on. They genuinely, I genuinely don't think they realise the impact they have on mine and other people's lives here’ (P10, female).  ‘I can help someone who has just walked through the door and remember that I felt like and reassure them that yeah it's scary, but you're here, you belong’ (P11, male).  ‘They're in front of me doing everything I'm trying to do’ (P14, female).  ‘I just think if there's other people here dealing with other things and still managing to do it, why can't I just follow what they're doing?’ (P15, male).  ‘Knowing that there's people here going through life's difficulties and not using, that inspires me and shows me that there's another way’ (P15, male). |
| Experiencing life outside of SUD |  | ‘It's funny because I've not cared one bit before about that, like the environment, I've got bigger fish to fry when I'm stuck trying to score, right? But now it feels like refreshing and it's cool that I'm interested in that’ (P2, male).  ‘It's a way to be a productive member of society’ (P3, male).  ‘They're like oh my god I can cook, I can make this, I can give something back, I can do a job and I can complete a task and a lot of people haven't had that before so it's definitely huge’ (P6, female).  ‘this (planting flowers) just brings back memories, happy memories’ (P7, male).  ‘'it gives you a chance to reconnect with your past?' yeah but in a positive light, the really good bits, the bits you want to remember and the childhood members you love’ (P7, male).  ‘This is an opportunity of practising taking my recovery into other aspects of life, so that's like socialising, meeting new people, looking at employment, do you know, all that other stuff’ (P9, female).  ‘This helps with a fulfilling life’ (P9, female).  ‘By making you feel normal, like you don't have a group to have a laugh with in active addiction, you know, you're isolated and your routine revolves around the dealer, but here you get a taste of what life should be’ (P10, female).  ‘It teaches me how to be a productive member of society and how to like experience joy I suppose, find things I like because you don't get that in active addiction’ (P10, female).  ‘Some sort of, yeah, identity. I think Getting Clean has helped me to find that in’ (P11, male).  ‘I'm slowly finding myself again’ (P13, female).  ‘This gives me, like outside of the meetings, this gives me a purpose, you can find out what your skills are’ (P14, female).  ‘It's getting me ready for the outside world’ (P14, female).  ‘Stuff like this helps me feel like I'm being the real genuine me, the person I was meant to be before I use, before addiction’ (P15, male).  ‘This life now, that's a life beyond my wildest dreams from all those years in addiction’ (P15, male). |
| Changing public perspective of addiction |  | ‘I think when people start looking deeper into it and seeing a person behind that, the idea seems to change a little bit’ (P1, male).  ‘Where she saw you scoring at the bottom of the road she's now seeing you pick her rubbish up and that must be cool, right?’ (P1, male).  ‘And the community also learn and hopefully see us for the good we're doing’ (P1, male).  ‘I could tell you that I've been to prison but I'm a good person, but would you believe someone saying that? I don’t know, but I could show you the good I'm doing by cleaning up and going to markets and prove that’ (P1, male).  ‘Hopefully do good and change people's thoughts on addicts too. People can see, yeah, I was an addict but I'm looking to change my life and do good’ (P2, male).  ‘Hopefully giving people a different perspective on addiction and what recovery looks like too’ (P7, male).  ‘There's a lot of negative stereotyping around addiction and stuff like that, so if people who don't know details about it can see us giving back it gives them a different point of view’ (P7, male).  ‘We went and explained who we are and what we do and they said they wouldn't have ever guessed this was people in recovery’ (P7, male).  ‘There's so much stigma around addiction but from this we're getting to speak to people and change their image of addiction’ (P10, female).  ‘Some people can look down on us but this shows that we're all human beings, we've all got issues, but we really can correct ourselves and do good for other people’ (P13, female). |
| Psychological impact |  | ‘It felt good, this place makes me feel good about myself’ (P2, male).  ‘I feel listened to and heard here’ (P2, male).  ‘It feels like I'm normal, like a member of society doing things I enjoy’ (P4, male).  ‘Here the level of care is just above and beyond. It's sometimes weird for my mean brain but over time I've learned to get used to it’ (P10, female).  ‘You do have like a voice here, you do, you're listened to and like I said before, that matters when you might not have had that before’ (P10, female).  ‘The fact there's hope, the hope side of it, because when you're an addict you lost all forms of hope’ (P10, female).  ‘It gives me a purpose, and meeting and being around other people, I go away feeling better about myself’ (P12, female).  ‘This gives me, like outside of the meetings, this gives me a purpose’ (P14, female).  ‘To be trusted with that (money), it feels amazing’ (P14, female).  ‘I've got a voice, I didn't used to have one, but I've got one now’ (P14, female). |
|  | Feeling a sense of belonging | ‘The only place I've ever felt a part of is here. Well, the other place is prison, and that's a mad perception, that's not right’ (P1, male).  ‘I'm so comfortable here and it feels like a safe space’ (P1, male).  ‘You can head and like spend time there and feel a part of. The best thing about Getting Clean are [the volunteers] because they just properly care about you’ (P3, male).  ‘As soon as I got here I was made to feel so included’ (P4, male).  ‘If you're like an anxious person or if you've been removed from society for a long time be it prison or be it just your own doings and so on, the public and reality seems to be a thing you observe and think of lots and something you don't feel a part of when you're in addiction... I now realised that you know, I am a part here, you're not invisible now because many people feel completely invisible, unseen, like nobody knows anything about them’ (P4, male).  ‘They are supporting me just, just getting through day by day at the moment’ (P6, female).  ‘It's just nice to know where I'm welcomed’ (P6, female).  ‘I feel like I'm a part of something’ (P7, male).  ‘I can be myself, I don't have to put on a face. I've, I've lived my life wearing different masks, you know, my work mask, my mum mask, my wife mask, I feel like I can just be myself and bring it all to this place’ (P9, female).  ‘By making you feel normal, like you don't have a group to have a laugh with in active addiction, you know, you're isolated and your routine revolves around the dealer, but here you get a taste of what life should be like with that fun and support and doing things for the wider community’ (P10, female).  ‘For a few hours on a Friday and makes you feel normal and liked’ (P10, female).  ‘You're here, you belong’ (P11, male).  ‘They can come here and find a sense of belonging’ (P11, male).  ‘It's nice that you feel part of something, it is, it brings a lovely feeling’ (P11, male).  ‘Family don't understand, and you can just be you’ (P12, female).  ‘I feel like, loved, as soon as I came up the steps today people were like ay! You're here! So yeah, it makes you feel really happy, it makes you feel wanted’ (P13, female).  ‘You don’t have to hide behind a mask or anything, you know, no matter how you present yourself whether that's good or bad’ (P13, female).  ‘I was made to feel a part of something, made to feel valued, part of a team, a family I suppose. You know, all the connections, that I mean, that I'd never had my whole life’ (P15, male).  ‘I enjoy coming and I've finally found somewhere that I belong, where I fit in, I'd never felt like that ever in my life’ (P15, male). |
|  | Adopting a positive and rational mindset | ‘Here they're good at focussing on the now and doing what you can do now to be able to say look this happened, and I can't change that, but I can show you these things I've been doing now that I'm dead proud of’ (P1, male).  ‘The openness stands out here and a like positive stance, that yeah you've done a lot of bad things, but that's past tense’ (P4, male).  ‘Being involved here, it's like, I realised that I still had them skills and my time hadn't been wasted’ (P9, female).  ‘Just having that hope and this uplift, sometimes that's kept me from relapsing’ (P10, female).  ‘A group not like proud of our past, but just accepting, okay that happened but look what we're doing now’ (P10, female). |
|  | Improved self-worth | ‘People have their eyes open here in terms of what they can actually like do’ (P1, male).  ‘There's a lot of people who don't realise the skills that they have because it's been, it's been lost in a mire of years of hurt and abuse. And this place here, this place can start to build up, build up that self-esteem and show them that they have a worth’ (P1, male).  ‘It shows me that I'm decent and I can do good things. Yeah, that goes a long way.’ (P1, male).  ‘I don't think I would've spoken to anybody then because my self-esteem and how I treated myself was so low I would have presumed that even though you came here today and explained what you were doing, I would presume, no she doesn't mean me, nobody wants to hear from me’ (P1, male).  ‘Again the self-esteem part is huge, and it's like building up somebody's self-worth because you just don't think you've got a fucking worth to life’ (P1, male).  ‘I feel useful and like I can use what I know. And I feel busy and useful and that's always a good thing’ (P3, male).  ‘For others with nothing, no job experience, no skills, no knowledge of how to behave, for them it's so much more and it's that confidence. It just makes addicts feel useful and that's so important’ (P6, female).  ‘I lost confidence in myself and I think being involved here, it's like, I realised that I still had them skills and my time hadn't been wasted’ (P9, female).  ‘I think it's helped bring my confidence back, it's helped me learn who I am, you know? Because drugs kind of took all that away’ (P11, male).  ‘It's helped me to find confidence in myself and I know there's certain things I'm good at now. Say, I know I'm a good team player, teamwork, I can do that, and I can help people here’ (P11, male). |
| Skills acquisition |  | ‘On a Saturday we do a market stall selling soap and that's like keeping me busy but also working and learning and meeting new people and learning skills like I said’ (P3, male).  ‘I've definitely learned here to ride out how you feel’ (P4, male).  ‘I'm achieving things that six months ago … I was convinced that I would just have to stay on benefits and never be able to do that?’ (P9, female).  ‘Today I've taken a little bit of leadership’ (P11, male).  ‘I used to do things against my will, but now if I don't want to do something I just say no. And I've learned that no is a full sentence, you don't need to explain why, it's just no’ (P14, female).  ‘Seeing other people being like that, open, that's made me think if they can do it, I can do it’ (P15, male). |
|  | Practical skills | ‘The tier system being set up and the stuff to put on your CV, because you haven't had nowt. There's just a big gap on there, like a massive black hole on paper and employers see that and are like well where the fuck have you been for the last 30 years?’ (P1, male).  ‘Learning skills that are good for my future , like to me it all goes … to when I leave day care and I'm out in the real world. I'm learning skills here that I can transfer into life and transfer into jobs in the future’ (P3, male).  ‘People getting employed from it, those skills, that's cool. That's something I'm excited about because as I said I am so keen to work again’ (P5, female).  ‘They're like oh my god I can cook, I can make this, I can give something back, I can do a job and I can complete a task and a lot of people haven't had that before so it's definitely huge’ (P6, female).  ‘I can put [name] down as a reference!’ (P7, male).  ‘People have that CV stress too - what do I say about this time off?’ (P9, female).  ‘I've realised that I'm quite a practical person as well, like you know, I can make soap now.’ (P11, male). |
|  | Social and emotional skills | ‘Everyone talks here. This has like changed my social boundaries and skills. I didn't think I had social skills to get through to people because of where I'd been’ (P1, male).  ‘When you've been isolating, to communicate with people, that's massive. Like, imagine asking me a year ago to even come talk to you, Dr Gabby! No seriously, I don't think I would've spoken to anybody then’ (P1, male).  ‘My relationship with my family has massively improved since doing this’ (P1, male).  ‘I feel like this has sort of brought me out of that and pushed me, like I was never like this before, speaking to people and like you know, engaging with them’ (P4, male).  ‘When I came into recovery I came straight out of prison so I didn’t know how to socialise you know, I'd lost all of them skills, and Getting Clean has enabled me to get them back’ (P7, male).  ‘The impact on my daughter… for her to see me going out the house most days, sticking to my commitments. That's role modelling the things she's struggling to deal with’ (P9, female).  ‘Even for my daughter, I do, I do feel like I've gained more understanding in her mental health. I've been more understanding and a better mother’ (P9, female).  ‘My mum and dad love that I'm here’ (P10, female).  ‘When you come into recovery you need to learn to socialise, you need to learn to be around people’ (P14, female).  ‘I'm learning social skills doing this, like I'm going with [name] to speak to professionals, sitting in there, taking part, I could never do any of that before’ (P14, female).  ‘The first thing I said to them was 'can you put me in the store room because I'm not a people person?' … I'll speak to anyone now!’ (P14, female).  ‘I'm learning how to be a mum now’ (P14, female). |
|  | Insight into behaviour | ‘My brain has weird ways to do things and what I think is right I have to break down and work out and question’ (P1, male).  ‘I've gotten better at thinking about my emotions and like breaking things down in my head’ (P4, male).  ‘Even for my daughter, I do, I do feel like I've gained more understanding in her mental health’ (P9, female).  ‘The struggle now, it's gonna happen’ (P12, female).  ‘I'm very self aware now, I never knew any of that before’ (P14, female).  ‘I'd feel like using was the only way out and for ten seconds it was, like, it probably would bring a bit of relief but literally for about ten seconds and then I'd come pounding back’ (P15, male). |
| Fun |  | ‘We had a great day, a really good day. It felt fun and silly, but we were still doing good. And the fun-ness, that's what I was missing’ (P2, male).  ‘Here is more of a laugh, do you know what I mean? I actually enjoy it’ (P3, male).  ‘I like it, yeah, I didn't know if I would, or like I think I was trying to understand why were were doing it, then someone today they, they said you don't need to. Like see it as fun. That's what it is’ (P5, female).  ‘It can all be quite serious at times so it can be nice to do something for recovery that is just different to what we're doing day in day out’ (P6, female).  ‘When you're in meetings you're in a serious headspace but when you're out here it's a bit more relaxed and a bit more informal’ (P7, male).  ‘I've been making soap and I must say, I must whisper that I did enjoy it but you can't tell [friend]. He can't be right!’ (P8, female).  ‘Yeah it has felt fun and like, like refreshing. I suppose a bit childlike but maybe in a good way’ (P8, female).  ‘By making you feel normal, like you don't have a group to have a laugh with in active addiction, you know, you're isolated and your routine revolves around the dealer, but here you get a taste of what life should be like with that fun and support and doing things for the wider community’ (P10, female).  ‘It's just having a laugh and a joke and you go away, you can come in this room and feel the weight of the world on your shoulders… that fun stuff, and just having that hope and this uplift, sometimes that's kept me from relapsing’ (P10, female).  ‘We come here and we have a laugh and that in turn helps us with recovery’ (P10, female).  ‘This is for me, a bit of escapism to get away from that, cos that, that's just too much’ (P12, female).  ‘And by the time I leave, I'll feel better, I'll have had a laugh here, you know, they'll have lifted my spirits’ (P12, female).  ‘There's loads of people who come, we get involved with everything and have a laugh’ (P13, female).  ‘We’ve had a laugh today, you know, it’s not all about recovery’ (P13, female).  ‘It's just like watching people when they first come in, they've got no confidence, they've no, no social skills really just like I didn't, and seeing that light come back on in their eyes, seeing them enjoying what they're doing, just seeing them laugh, laughter!’ (P14, female). |
| Feeling supported in recovery |  | ‘I find that when I'm here there's always really good talk. It always tends to be recovery focussed, erm yeah, but it can be anything. It can be a really deep chat or what you're watching on TV’ (P2, male).  ‘I love doing the activities but this place is so much more. I think it's the mood side of things… I came for the activities at first but I stay because of the support’ (P2, male).  ‘Truly there's always emotional support there, always, you've always got that’ (P4, male).  ‘It’s just keeping me going at the moment, it really is’ (P6, female).  ‘I don’t know what I'd do without it, or I do…’ (P6, female).  ‘I do feel truly supported here’ (P6, female).  ‘When I was struggling this place was here, this place is always here… you've always got a safe place you can go every Friday’ (P10, female).  ‘Now I'm in rehab but I still do this because, because, this is what's kept me in a safe place when I was out there and when I was struggling’ (P10, female).  ‘She gave me the exact advice I needed. It was like she understood me better than I did because she's on the other side of it’ (P10, female).  ‘You can talk to people about what's going on with you, and they've all been through similar things so you know, you can like, so, you can hear their thoughts and get a different perspective on something’ (P12, female).  ‘I know that there's solutions now, solutions now to help you, you know, you don’t have to tackle life on your own’ (P12, female).  ‘They're here to listen to you, here to help you if they can’ (P13, female).  ‘We’re like a family here. They love you, they give you support. I feel like we could, well, not handle anything, but you know, you know they've got your back’ (P13, female).  ‘You know you can ring anyone here at any time and they we're always there for each other’ (P14, female). |
|  | Flexibility and trust in service users | ‘I said I can't commit to this for a while and they said listen, that's alright, we kind of knew that, just come back when you can and when you're ready and nothing will have changed’ (P2, male).  ‘That chilled environment? Yeah which sounds basic but, but it's not. It means a lot and it feels respectful and truly caring’ (P2, male).  ‘It is flexible, it seems er very easy and I need that, easy going and okay with people saying no’ (P8, male).  ‘they've said her how much do you want to be involved, right, what can you bring in? How do you want to go about that? And then we've kind of aligned’ (P9, female).  ‘It still allows me to be me as well as a mum’ (P9, female).  ‘Come here every day, or whenever you want’ (P10, female).  ‘You can volunteer as much or as little as you want here’ (P14, female). |
|  | Dependable and consistent sessions | It's don’t rush, take your time, there's no end date, and it's patient here, there's so much patience for everyone’ (P2, male).  ‘It was there when I needed it and it's there for other people when they need it, whatever the time’ (P7, male).  ‘When I was struggling this place was here, this place is always here… you've always got a safe place you can go every Friday’ (P10, female).  ‘It's every week at the same time and in the same place so even if you haven't got a phone, you haven't got anywhere to live, you know, you can even go downstairs and have a shower’ (P10, female).  ‘No matter what you've got going on, this is a constant. If you've lost your phone you know where it is, if you've been kicked out of your programme you're still welcome here’ (P10, female).  ‘This place, always here, come whenever, no judgement’ (P10, female).  ‘No one is going to kick you out the door… the support is always there, that's the main thing, the support is always there for you’ (P11, male).  ‘You know you can ring anyone here at any time and they we're always there for each other’ (P14, female). |
| Preventing relapse by filling time |  | ‘When I've got time on my hands that's a problem, that's when problems start’ (P1, male).  ‘Especially when you're in recovery, and early recovery, sometimes its not good to be sat around with your head’ (P1, male).  ‘Coming here, at first it was to occupy my time so I wasn't sat there in my own bed thinking about using. I had to do something to get out of that’ (P1, male).  ‘I can use the example of youth clubs. It's like youth clubs for kids’ (P1, male).  ‘I kept doing litter picking because it gave me sommat to do’ (P2, male).  ‘I was keeping myself busy but even then I thought what am I gonna do with my time? What am I gonna do with all my time now I'm not doing group sessions? And you know, I thought, do what I did before, Getting Clean, that kept me busy and that made me feel good. Lets go back there’ (P2, male).  ‘Coming here is really good. It does keep me busy. I need those skills and I need to keep busy because I was busy before and during a lot of my drug use’ (P2, male).  ‘I come to a space out of the hours of my daycare, and especially on the Fridays, some people struggle with Friday's because people get that Friday feeling’ (P3, male).  ‘I think I'd have been closer to relapsing if I didn't fill my weekends’ (P3, male).  ‘It's the weekends that are lots of time for you and I was worrying about that, like for me time isn't a good thing really. So I was worried about how I was gonna fill my time then my housemate said that he was off to make soap and I followed him down and ended up in one of the sessions on a Friday’ (P4, male).  ‘It's just keeping me busy at the moment’ (P6).  ‘I've been employed most of my life… for me it's good to keep busy’ (P6).  ‘It's something extra to do during the day’ (P7, male).  ‘Keep myself busy cos like I say I've worked for the past eight years, so I’ve gone from 8 to 16 hour shift days to nothing. Too much bloody time do you know what I mean?’ (P8, male).  ‘Too much extra time and my mind runs and that's when it gets that overwhelming, back to that overwhelming feeling’ (P8, male).  ‘The routine is you get up, go see your dealer, and that's even if you've slept, go see your dealer and that's it, that's your only form of routine, but instead, this place is instilling a routine for people. That routine of giving people a place to go and something to do that doesn't involve that cycle’ (P10, female).  ‘On some Fridays I've been ready to relapse and here, from going here on a Friday, it's kept me from relapsing’ (P10, female).  ‘Here breaks the cycle of dealer using dealer using’ (P10, female).  ‘If I'm sat at home I'm just thinking and doing nothing if you know what I mean, so I'll just be sleeping and by the time the days ended I feel ever worse because I haven't done owt’ (P12, female).  ‘This morning I thought to myself, do you know what, I just wanna go score, but I knew this was on and I phoned a friend who said she was coming and I though get yourself down here, even though I didn't really wanna, I just thought I'd get myself out and down here and it'll kill a few hours, you know? And by the time I leave, I'll feel better, I'll have had a laugh here, you know, they'll have lifted my spirits’ (P12, female).  ‘It helps me with those thoughts you know… I finish group at 12 and then don’t go to a meeting till 6:30 so it's a long time to be with yourself’ (P13, female).  ‘It's better than being sat at home and not doing anything … those couple of hours at home, my brain just, it just thinks about using’ (P13, female).  ‘It's a nice place to be, and not forget everything, but to take a break from it, from all your struggles, and get on with something that distracts you from that, from all your struggles and the bad stuff that goes on’ (P13, female).  ‘It's okay going to a meeting but they only last like an hour, an hour and a half, so this gives you other things to do in your day’ (P14, female). |
