# Supplementary material for: Experiences of a lived experience recovery organisation for those in abstinence-based substance use recovery: a thematic analysis
Source: Subst Abuse Treat Prev Policy. 2025 Oct 9;20:41. doi: 10.1186/s13011-025-00671-9 (PMC12512285; doi:10.1186/s13011-025-00671-9)
Supplement: Supplementary file 2 — Supplementary Material 2. [file 13011_2025_671_MOESM2_ESM.docx]

**Consolidated criteria for reporting qualitative studies (COREQ): 32-item checklist**

| **No. Item** | **Guide questions/description** | **Reported on Page #** |
| --- | --- | --- |
| **Domain 1: Research team and reﬂexivity** |  |  |
| *Personal Characteristics* |  |  |
| 1. Interviewer/facilitator | Which author/s conducted the interview or focus group? | Both GH and NF conducted these interviews.  Reported on page 11 |
| 2. Credentials | What were the researcher’s credentials? E.g. PhD, MD | GH holds a PhD, MSc, and BSc in psychology and research methods. NF holds a MSc, BSc and has been a registered mental health nurse in the area of addiction for over two decades.  Reported on page 1 |
| 3. Occupation | What was their occupation at the time of the study? | GH is a lecturer in psychology, NF is an assistant professor in mental health nursing.  Reported on page 1 |
| 4. Gender | Was the researcher male or female? | Both GH and NF are female.  Reported on page 11 |
| 5. Experience and training | What experience or training did the researcher have? | Both researchers have professional training in qualitative research methods, and interviewing participants in a trauma informed manner.  Reported on page 11 |
| *Relationship with participants* |  |  |
| 6. Relationship established | Was a relationship established prior to study commencement? | No participants knew researchers prior to the study taking place. NF was aware of Getting Clean LtD – the LERO examined in the current study – through experts by experience teaching events at the university.  Reported on page 9 |
| 7. Participant knowledge of the interviewer | What did the participants know about the researcher? e.g. personal goals, reasons for doing the research | Participants knew this was a research study conducted to examine the impact of LEROs in addiction services. They were asked to be honest about their experiences and they were made aware of the topics being discussed in interviews to allow for informed participation.  Reported on page 10 |
| 8. Interviewer characteristics | What characteristics were reported about the interviewer/facilitator? e.g. Bias, assumptions, reasons and interests in the research topic | White female researchers, both working as lecturers. One previously worked as a mental health nurse in the area of addiction.  Reported on page 12 |

| **Domain 2: study design** |  |  |
| --- | --- | --- |
| *Theoretical framework* |  |  |
| 9. Methodological orientation and Theory | What methodological orientation was stated to underpin the study? e.g. grounded theory, discourse analysis, ethnography, phenomenology, content analysis | Thematic analysis was used in the current study, with in inductive approach followed for coding of themes.  Reported on page 11 |
| *Participant selection* |  |  |
| 10. Sampling | How were participants selected? e.g. purposive, convenience, consecutive, snowball | This was convenience sampling, with participants who turned up to the LERO asked if they wanted to take part the following week.  Reported on page 10 |
| 11. Method of approach | How were participants approached? e.g. face-to-face, telephone, mail, email | Participants were approached face-to-face and then provided with printed resources.  Reported on page 10 |
| 12. Sample size | How many participants were in the study? | 15  Reported on page 9 |
| 13. Non-participation | How many people refused to participate or dropped out? Reasons? | No participants refused to participate or dropped out from the current study.  Reported on page 12 |
| *Setting* |  |  |
| 14. Setting of data collection | Where was the data collected? e.g. home, clinic, workplace | Data was collected in a private room within the community hall the LERO also operated in. This room was located at opposite ends of the building, with a closed door, and it was ensured before beginning interviews that noise didn’t travel.  Reported on page 10 |
| 15. Presence of non-participants | Was anyone else present besides the participants and researchers? | Only the participants and the researchers were present for interviews in a closed room, although all interviews were recorded for transparency.  Reported on page 10 |
| 16. Description of sample | What are the important characteristics of the sample? e.g. demographic data, date | 15 participants were recruited; 8 male, 7 female, with a mean age of 39.3 year and SD of 7.41.  Reported on page 12 |
| *Data collection* |  |  |
| 17. Interview guide | Were questions, prompts, guides provided by the authors? Was it pilot tested? | Semi-structured interview script is in the appendices.  Reported on page: appendices. |
| 18. Repeat interviews | Were repeat interviews carried out? If yes, how many? | Participants were only interviewed once by the research team.  Reported on page 10 |
| 19. Audio/visual recording | Did the research use audio or visual recording to collect the data? | Interviews were all audio recorded on a dictaphone.  Reported on page 10 |
| 20. Field notes | Were ﬁeld notes made during and/or after the interview or focus group? | Field notes were not made during interviews, ensuring participants felt listened to. Notes were made afterwards on any suggested adaptations to interviews,  Reported on page 11 |
| 21. Duration | What was the duration of the interviews or focus group? | Interviews lasted from 19 to 49 minutes in duration.  Reported on page 10 |
| 22. Data saturation | Was data saturation discussed? | Yes, and reached at 15 participants.  Reported on page 12 |
| 23. Transcripts returned | Were transcripts returned to participants for comment and/or correction? | No.  Reported on page 11 |
| **Domain 3: analysis and ﬁndings** |  |  |
| *Data analysis* |  |  |
| 24. Number of data coders | How many data coders coded the data? | Two people coded this data – GH and NF.  Reported on page 12 |
| 25. Description of the coding tree | Did authors provide a description of the coding tree? | Full description via thematic map and then discussed via the analysis section.  Reported on page 13 onwards |
| 26. Derivation of themes | Were themes identiﬁed in advance or derived from the data? | An inductive approach was followed, meaning no themes were identified in advance of coding the data.  Reported on page 11 |
| 27. Software | What software, if applicable, was used to manage the data? | NVivo was used to code the data.  Reported on page 12 |
| 28. Participant checking | Did participants provide feedback on the ﬁndings? | Participants have not provided feedback on the findings. Although, participants have been sent a copy of the thematic map as requested – to highlight the impact of this group in a LERO session.  Reported on page 12 |
| *Reporting* |  |  |
| 29. Quotations presented | Were participant quotations presented to illustrate the themes/ﬁndings? Was each quotation identiﬁed? e.g. participant number | Yes  Reported on pages 12 onwards |
| 30. Data and ﬁndings consistent | Was there consistency between the data presented and the ﬁndings? | We feel data was presented with transparency and consistency.  Reported on page 12 onwards |
| 31. Clarity of major themes | Were major themes clearly presented in the ﬁndings? | We feel themes were presented clearly.  Reported on page 12 onwards |
| 32. Clarity of minor themes | Is there a description of diverse cases or discussion of minor themes? | We feel that data was incredibly rich in all areas and no themes took a backseat. Equally, data was very uniformed with no real anomaly in discussion.  Reported on: N/A |
